# EVIPNet Americas: Overcoming barriers to integrate scientific evidence and health policies in the Americas

**DOI:** 10.1016/j.lana.2022.100299

**Published:** 2022-06-14

**Authors:** Cristián Mansilla, Evelina Chapman, Jorge Barreto, Tomás Pantoja, Michelle Haby, Marina Melo Arruda Marinho, Daniela Fortunato Rêgo, Lucy Kuhn-Barrientos, Rocío Bravo-Jeria, Carolina Castillo, Carmen Verônica Mendes Abdala, Sebastian García-Saiso, Ludovic Reveiz

**Affiliations:** a Department of Evidence and Intelligence for Action in Health, Pan American Health Organization, Washington, DC 20037, USA; b Oswaldo Cruz Foundation, Brasília, Brazil; c Department of Family Medicine, Pontificia Universidad Católica de Chile, Chile; d Departamento de Ciencias Químico-Biológicas, Universidad de Sonora, México; e Department of Science and Technology, Ministry of Health, Government of Brazil, Brasilia, Brazil; f Unit of Evidence-Informed Health Policies, Department of Health Technology Assessment and Evidence-based Health, Ministry of Health, Government of Chile, Santiago, Chile; g Incident Management System for COVID-19, Pan American Health Organization, Washington, DC 20037, USA

In response to the challenges created by the COVID-19 pandemic to the role of evidence in policymaking, the World Health Organization (WHO) in collaboration with the Pan American Health Organization (PAHO) convened the first Global Evidence-to-Policy (E2P) Summit. This summit brought together different stakeholders from all WHO Regions to identify common challenges, share lessons learned, and provide recommendations to support evidence-informed decision-making as a catalyst for policy and societal changes.

The E2P summit was organized by the Evidence-Informed Policy Network (EVIPNet), a global initiative with strong experience in promoting the use of evidence in policymaking.^1^ In the Americas, EVIPNet has supported countries since 2007 in informing decisions with scientific evidence, with active long-lasting commitments from different countries,^2^ including Brazil^3^ and Chile.^4^

As part of the Summit, PAHO convened an EVIPNet Americas regional meeting to discuss the processes for using scientific evidence for the COVID-19 pandemic response and the challenges faced in translating this knowledge into policies in Latin America and the Caribbean (LAC). The event contributed to strengthening the EVIPNet Americas network. Three different panels were convened that provided insights into the use of evidence during the COVID-19 pandemic, experiences of Ministries of Health, and shared strategies and mechanisms for institutionalizing the use of evidence. The panels and participants shared important insights and lessons from different institutions in different countries (including Argentina, Brazil, Colombia, Chile, Ecuador, El Salvador, Mexico, and Peru). They reflected on several experiences of encouraging the use of evidence by public institutions to effectively respond to the COVID-19 pandemic, such as the need to better communicate evidence, to strengthen the relationship between evidence producers and decision makers, and for greater capacity-building in non-pandemic times to ensure better preparedness.

EVIPNet Americas and PAHO have used their experience in supporting LAC countries to encourage collaboration between stakeholders and to use evidence better in responding to the COVID-19 pandemic (see Table 1). The E2P Summit created a global venue to share lessons and seize the momentum created by the COVID-19 pandemic. The EVIPNet Call for Action^5^ was launched during the Summit, explicitly inviting governments, intergovernmental organizations, funders and other key stakeholders to join EVIPNet and commit to 16 concrete steps towards better evidence-informed decision-making. The critical insights and lessons learned from the acute phase of the COVID-19 pandemic will help EVIPNet Americas to better support countries in LAC to close the gap between evidence and decision-making processes.

**Table 1:** Resources and activities provided by EVIPNet Americas and the Pan American Health Organization to support evidence-informed decision-making.

Name of the resource/activity	Description	URL
**Guidance and methodological support**		
A Guide for Evidence-Informed Decision-Making, Including in Health Emergencies	This guide aims to bring together the most recent thinking in the Evidence-Informed Decision-Making (EIDM) field and to present this in a format that is accessible to a wide audience of EIDM practitioners.	https://iris.paho.org/handle/10665.2/55828
Strengthening national evidence-informed guideline programs. A tool for adapting and implementing guidelines in the Americas	Policy-oriented and methodological strategies for developing and/or strengthening national guideline programs, focusing on the adaptation of evidence-informed guidelines in the Americas.	https://iris.paho.org/handle/10665.2/49145
EVID@Easy	A guided search tool in the Virtual Health Library (VHL). EVID@Easy offers the trails to locate the scientific evidence available in the VHL according to the stage of the decision-making process: • Understanding the health problem • Identifying and selecting options to face the problem • Analyzing aspects and considerations for implementing options • Monitoring and evaluating the impact of the (decision) implementation	https://bvsalud.org/evideasy/en/
**Online courses and training programs**		
Introductory course on evidence-informed policies	Online self-learning course available Portuguese and Spanish	https://www.campusvirtualsp.org/es/node/31307
Introductory course on developing and adapting Guidelines using the GRADE Methodology	Online self-learning course available in English and Spanish on guidelines using GRADE.	https://www.campusvirtualsp.org/en/course/introductory-online-course-developing-and-adapting-guidelines-using-grade-methodology
**Databases and repositories**		
BIGG-REC	A database incorporating up-to-date WHO/PAHO evidence informed recommendations	https://bigg-rec.bvsalud.org/
BIGG	An international database of GRADE guidelines	https://sites.bvsalud.org/bigg/
PAHO Evidence and Intelligence for Action on the SDG-3 Targets	Portal improving access to data, analysis on health inequalities and evidence on interventions for the Sustainable Development Goal (SDG) 3.	https://www3.paho.org/ods3/
VHL Windows of Knowledge – Evidence-Informed Policy (EIP)	Collection of pre-made searches on the VHL database, along with links to materials and resources of the EIP methodology, aiding on the implementation and execution of this method.	https://bvsalud.org/vitrinas/en/post_vitrines/evidence-based-policy/
PIE – Evidence-Informed Policies	Database of documents that contribute to policy decision-making processes based on the best available scientific evidence from Latin America and the Caribbean Countries	https://sites.bvsalud.org/pie/
**Evidence syntheses**		
Ongoing Living Update of Potential COVID-19 Therapeutics Options: Summary of Evidence. Rapid Review	Living evidence synthesis on the benefits and harms of therapeutic options against COVID-19	https://iris.paho.org/handle/10665.2/52719
PAHO evidence synthesis collection	The evidence syntheses and recommendations, published by the Pan American Journal of Public Health, aim to present the evidence-informed recommendations developed by PAHO and WHO in a way that facilitates their dissemination, adaptation and implementation in the countries of the Region	https://www.paho.org/journal/en/special-issues/pahowho-evidence-synthesis-and-recommendations
Examples of country-level resources*		
Brazil	Online course on evidence-informed policies, that is offered in partnership with the Hospital do Coração (HCOR)	https://ead.hcor.com.br/blog/index.php?entryid=4
	Methodological guideline: evidence synthesis for policies	https://bvsms.saude.gov.br/bvs/publicacoes/diretriz_sintese_evidencias_politicas.pdf
Chile	Methodological handbook on how to produce rapid-evidence syntheses	https://etesa-sbe.minsal.cl/wp-content/uploads/2018/06/PDF-1-Manual-Metodol%c3%b3gico-EVIPNet-Chile.pdf
	Evidence map of COVID-19 research to inform decision-making	https://diprece.minsal.cl/wp-content/uploads/2020/08/evidence-map-covid-19-v8.html

⁎ This is not a full list of resources that countries from the Americas have made available.

## Contributors

Conceptualization: L.R., C.M., J.B. and T.P., Writing – original draft: C.M., E.C., J.B., T.P., M.H., AND L.R., Writing – review & editing: M.M., D.F., L.K., R.B., C.C., C.V.M.A., and S.G. All authors reviewed and agreed with this final version of the paper.

## Data sharing statement

No individual data was used for this manuscript. The resources mentioned are all publicly available under the URLs reported in the table.

## Funding

This commentary did not have any source of funding.

## Disclaimer

The author is a staff member of the Pan American Health Organization. The author alone is responsible for the views expressed in this publication, and they do not necessarily represent the decisions or policies of the Pan American Health Organization.

## Declaration of interests

The authors have declared that no competing interests exist. Authors hold sole responsibility for the views expressed in the manuscript, which may not necessarily reflect the opinion or policy of the Pan American Health Organization.
